# Body position during transport in a refractory cardiac arrest porcine model

**DOI:** 10.1186/cc14497

**Published:** 2015-03-16

**Authors:** J Belohlavek, M Mlcek, M Huptych, T Boucek, T Belza, P Krupickova, O Kittnar

**Affiliations:** 1 General University Hospital, Prague, Czech Republic; 2 Charles University in Prague, Czech Republic; 3 Czech Technical University in Prague, Czech Republic

## Introduction

Cardiac arrest patients are not transported only supine. The effect of body position on resuscitability and cerebral perfusion in a 30° and 60° incline is not known.

## Methods

Twenty-five female pigs were subjected to a simulated cardiac arrest (3 minutes no flow, 5 minutes mechanical CPR). Next, animals were randomly assigned to one of the three groups: GROUP 60 (*n *= 8), 60° incline for 3 minutes to simulate transport in space restricted elevator; GROUP 30 (*n *= 8), 30° incline for 8 minutes to simulate staircase transport; and GROUP 0, with no incline. During subsequent standard CPR including rescue ECMO, resuscitability and cerebral perfusion were assessed.

## Results

**Figure 1:**
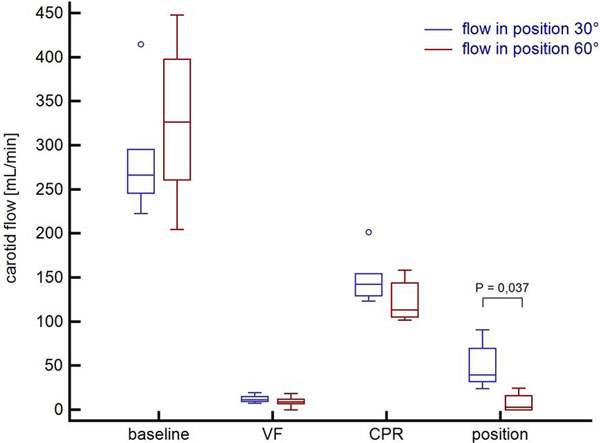


Attainment of ROSC (3, 5, 5 in respective groups, *P *= 0.021), time to ROSC (15:24 (13:26; 16:02) vs. 19:19 (18:28; 19:37) vs. 9:10 minutes (8:28; 9:41), respectively, *P *= 0.005) significantly differed. Changes in carotid blood flow according to the respective periods of the protocol (baseline, cardiac arrest, initial supine CPR and 30° vs. 60° CPR) are depicted in Figure 1.

## Conclusion

Positional changes during simulated refractory cardiac arrest in this experimental model significantly affect resuscitability and brain perfusion. Animals subjected to shorter time in a more inclined (GROUP 60) position were more easily resuscitated; however, cerebral blood flow was better preserved in GROUP 30.

